# Italian real life experience with brentuximab vedotin: results of a large observational study on 234 relapsed/refractory Hodgkin’s lymphoma

**DOI:** 10.18632/oncotarget.18114

**Published:** 2017-05-23

**Authors:** Cinzia Pellegrini, Alessandro Broccoli, Alessandro Pulsoni, Luigi Rigacci, Caterina Patti, Guido Gini, Donato Mannina, Monica Tani, Chiara Rusconi, Alessandra Romano, Anna Vanazzi, Barbara Botto, Armando Santoro, Stefan Hoaus, Gian Matteo Rigolin, Pellegrino Musto, Patrizio Mazza, Stefano Molica, Paolo Corradini, Angelo Fama, Francesco Gaudio, Michele Merli, Fioravante Ronconi, Giuseppe Gritti, Daniele Vallisa, Patrizia Tosi, Anna Marina Liberati, Antonello Pinto, Vincenzo Pavone, Filippo Gherlinzoni, Maria Paola Bianchi, Stefano Volpetti, Livio Trentin, Maria Cecilia Goldaniga, Maurizio Bonfichi, Amalia De Renzo, Corrado Schiavotto, Michele Spina, Angelo Michele Carella, Vittorio Stefoni, Lisa Argnani, Pier Luigi Zinzani

**Affiliations:** ^1^ Institute of Hematology “L. e A. Seràgnoli”, University of Bologna, Bologna, Italy; ^2^ Department of Cellular Biotechnologies and Hematology, Hematology, Sapienza University, Rome, Italy; ^3^ Department of Hematology, University and Hospital Careggi, Firenze, Italy; ^4^ Department of Hematology, Azienda Ospedali Riuniti Villa Sofia Cervello, Palermo, Italy; ^5^ Department of Hematology, Ospedali Riuniti di Ancona, Ancona, Italy; ^6^ Hematology Unit, Ospedale Papardo, Messina, Italy; ^7^ Hematology Unit, Santa Maria delle Croci Hospital, Ravenna, Italy; ^8^ Division of Hematology, Niguarda Hospital, Milan, Italy; ^9^ Division of Hematology, AOU, Catania, Italy; ^10^ European Institute of Oncology, Milano, Italy; ^11^ Hematology, Azienda Ospedaliera Universitaria Città della Salute e della Scienza, Torino, Italy; ^12^ Oncology and Hematology, Humanitas Cancer Center, Rozzano, Italy; ^13^ Institute of Hematology, Catholic University, Rome, Italy; ^14^ Hematology Section, St. Anna University Hospital, Cona, Ferrara, Italy; ^15^ Scientific Direction, IRCCS-CROB, Referral Cancer Center of Basilicata, Rionero in Vulture, Pz, Italy; ^16^ Ospedale Moscati, Department of Hematology-Oncology, Taranto, Italy; ^17^ Department of Hematology, Ciaccio-Pugliese Hospital, Catanzaro, Italy; ^18^ Department of Hematology, Fondazione IRCCS Istituto Nazionale dei Tumori, University of Milan, Milan, Italy; ^19^ Hematology Unit, Arcispedale Santa Maria Nuova, Istituto di Ricovero e Cura a Carattere Scientifico, Reggio Emilia, Italy; ^20^ Hematology Unit, Policlinico di Bari, Bari, Italy; ^21^ Hematology, Ospedale di Circolo, Fondazione Macchi, Varese, Italy; ^22^ Division of Hematology and Stem Cell Transplantation Unit, Cardarelli Hospital, Napoli, Italy; ^23^ Department of Hematology, Hospital Papa Giovanni XXIII, Bergamo, Italy; ^24^ Division of Hematology, Guglielmo da Saliceto Hospital, Piacenza, Italy; ^25^ Hematology Unit, Infermi Hospital Rimini, Rimini, Italy; ^26^ Hematology, Ospedale Perugia, Perugia, Italy; ^27^ Hematology-Oncology and Stem Cell Transplantation Unit, National Cancer Institute, Fondazione Pascale, IRCCS, Napoli, Italy; ^28^ Division of Hematology, Ospedale G. Panico, Tricase, Lecce, Italy; ^29^ Hematology Unit, Ca’ Foncello Hospital, Treviso, Italy; ^30^ Sant’Andrea Hospital, Sapienza University, Rome, Italy; ^31^ Department of Hematology, DISM, Azienda Sanitaria Universitaria Integrata, Udine, Italy; ^32^ Department of Medicine, Hematology and Clinical Immunology Unit, University of Padua, Padua, Italy; ^33^ Onco-Hematology Unit, Fondazione Ca’ Granda IRCCS Ospedale Maggiore Policlinico, Milan, Italy; ^34^ Hematology, IRCCS Policlinico San Matteo, Pavia, Italy; ^35^ Hematology, AOU Federico II Napoli, Napoli, Italy; ^36^ Hematology, San Bortolo Hospital, Vicenza, Italy; ^37^ Division of Medical Oncology A, National Cancer Institute, Aviano, Italy; ^38^ Division of Hematology 1, IRCCS A.O.U. San Martino IST, Genova, Italy

**Keywords:** brentuximab vedotin, long-term response, real life, Hodgkin’s lymphoma, stem cell transplantation

## Abstract

A large Italian multicenter observational retrospective study was conducted on the use of brentuximab vedotin (BV) for patients with relapsed Hodgkin’s lymphoma (HL) to check if clinical trial results are confirmed even in a real life context. 234 CD30+ HL patients were enrolled. Best response was observed after a median of 4 cycles in 140 patients (59.8%): 74 (31.6%) patients obtained a complete response (CR) and 66 (28.2%) achieved a partial response (PR); overall response rate at the end of the treatment was 48.3% (62 CR and 51 PR). The best response rate was higher in the elderly subset: 14 (50%) CR and 5 (17.8%) PR. Disease free survival was 26.3% at 3 years and progression free survival 31.9% at 4.5 years. Duration of response did not differ for who achieved at least PR and then either did or did not undergo consolidative transplant. Overall, the treatment was well tolerated and no death has been linked to BV-induced toxicity.

Our report confirms activity in elderly patients, duration of response unrelated to the consolidation with transplant procedure, the relevance of the CR status at first restaging, and the role of BV as a bridge to transplant for chemorefractory patients.

## INTRODUCTION

Conventional chemotherapy (with radiotherapy in case of localized disease) has made Hodgkin’s lymphoma (HL) a curable disease for most of the patients. Salvage chemotherapy followed by high dose therapy and autologous stem cell transplantation (ASCT) represents the treatment of choice for relapsed/refractory (R/R) patients, since this therapeutic strategy can provide long-term disease control in approximately 50% of R/R patients.[1-6] For patients with HL who relapse after ASCT, conventional salvage chemotherapies are often unsatisfactory. The outcome of this subset of patients is rather dismal with a median overall survival (OS) of 2 years.[7, 8] Relapsed disease after ASCT is considered incurable, unless allogeneic transplant (alloSCT) is applied. However, very few patients can achieve this goal, since refractory disease often hampers the benefits of this procedure.

CD30 is a transmembrane-receptor protein expressed on activated B and T lymphocytes and on the surface of malignant Hodgkin Reed-Sternberg cells. Through CD30-CD30-ligand interaction, eosinophils and mast cells may stimulate those malignant cells, promoting their survival.[9] Brentuximab vedotin (BV) is a chimeric anti-CD30 antibody conjugate via a protease-cleavable linker to a microtubule-disrupting agent, monomethyl auristatin E. After binding to cell-surface CD30, that agent is internalized, traffics to the lysosome and is released to disrupt microtubules, inducing cell-cycle arrest and apoptosis.[10] Several studies have shown the efficacy of BV in patients with HL starting from the pivotal phase II study on patients with R/R disease after ASCT, reporting a 75% of objective response rate (ORR) with 34% of complete response (CR).[11] This high response rate is important not only for heavily pretreated patients with a poor prognosis, but also for first-line R/R patients, because a CR status before the transplant procedure is one of the stronger predictors for long-term survival.[12-18] BV can represent an optimal therapeutic option as a bridge to ASCT or alloSCT in patients who showed a suboptimal response after conventional salvage treatment.[15, 19, 20] Recent updates of the two pivotal studies have shown that BV can induce long lasting CR in HL pretreated cases either without additional consolidation therapies suggesting that BV may lead to a long disease control in some patients.[21, 22]

After accelerated approval by US Food and Drug Administration, eligible patients in Italy were granted early access through a Named Patient Programme (NPP). After the closure of NPP, between 2012 and 2014 BV was available in Italy for patients with relapsed HL, based on a local disposition of the Italian Drug Agency issued according to a national law (Law 648/96: “medicinal products that are provided free of charge on the national health service”): a boundary zone in the passage from clinical trials to marketing and free use phases where patients can be treated in any case.

On the basis of our previous explorative study, a large Italian observational retrospective study was conducted on the use of BV in the everyday clinical practice to check if clinical trial results are confirmed even in a real life context. [23]

## RESULTS

Of the estimated 238 patients who received BV under the Law 648/96, 4 (2%) refused to participate in this observational study. Characteristics of the 234 patients are summarized in Table 1. The median age at BV was 35.4 years (range, 18-79 years) with 28 (12.0%) elderly patients (age≥ 60 years); 129 were males and 105 were females. One-hundred-sixteen (49.6%) had systemic symptoms at baseline.

**Table 1:** Patient demographics and characteristics at baseline

	Total population	Elderly (≥60)
Patients, *N*	234	28
Median age, years (range)	35.4 (18.0-79.0)	66.5 (60.2-78.6)
Median time from diagnosis-BV*, years (range)	2.3 (1.0-33)	2.9 (1.0-19.5)
Male, *N* (%)	129 (55.1)	17 (60.7)
Stage, *N* (%) - I/II - III - IV	99 (42.3) 48 (20.5) 87 (37.2)	11 (39.3) 6 (21.4) 11 (39.3)
ECOG^†^ performance status, *N* (%) −0 −1 −2	26 (60.5) 17 (39.5) -	7 (25.0) 16 (57.1) 5 (17.8)
Bulky disease, *N* (%)	12 (5.1)	1 (3.5)
Bone marrow involvement, *N* (%)	15 (6.4)	2 (7.1)
Systemic symptoms, *N* (%)	116 (49.6)	10 (35.7)
- Refractory to most recent therapy, *N* (%) - Refractory to first line therapy, *N* (%)	164 (70.1) 119 (50.8)	16 (57.1) 9 (32.1)
Median number of previous therapies (range)	3 (1-6)	2 (1-6)
Prior autologous stem cell transplant, *N* (%)	163 (69.8)	11 (39.3)
Prior radiotherapy, *N* (%)	98 (41.9)	7 (25.0)

^*^BV: brentuximab vedotin; ^†^ECOG: Eastern Cooperative Oncology Group.

The median number of prior treatment regimens was 3 (range, 1-6) including high dose chemotherapy and ASCT (in 163, 69.6% of the patients). Ninety-eight patients (41.9%) had received prior radiation therapy. For each patient the status after both frontline therapy and most recent therapy was collected: 119 (50.8%) patients had disease that was refractory to frontline therapy and 164 patients (70.1%) had disease that was refractory to last therapy before BV.

### Response to treatment

Best response was observed after a median of 4 cycles in 140 (59.8%) patients: 74 (31.6%) obtained a CR and 66 (28.2%) achieved a partial response (PR). ORR at the end of the treatment was 48.3% (113 patients) represented by 62 (26.5%) CR and 51 (21.7%) PR; among the remaining patients, 36 had stable disease (SD), and 85 patients showed progression of disease (PD), respectively.

The best response rate was higher (p<0.05) in the elderly subset (>60 years): 14 (50%) CR and 5 (17.8%) PR. In the elderly setting the ORR was 46.4% (13 patients) represented by 11 (39.3%) CR and 2 (7.1%) PR.

We performed analyses to report outcomes of patients who underwent stem cell transplant compared to those who did not. Patients who had a prior ASCT were further considered with two criteria: first we calculated overall response rate in patients who underwent ASCT any time before brentuximab then in the ones who had ASCT immediately before brentuximab. In patients who had ASCT any time before brentuximab (*n* = 163) ORR after BV was 53.9% and complete response rate 29.4%. In patients who had ASCT immediately before brentuximab (*n* = 89) ORR after BV was 75.3% and CR rate 29.2%. In patients who did not undergo ASCT before brentuximab (*n* = 71) ORR after BV was 50.7% and CR rate 25.4%.

Twelve patients who were in CR after 4 cycles (the first evaluation as per schedule) relapsed during subsequent BV courses; fifteen patients who were in PR at the first restaging converted to CR status after further four BV infusions. All patients but one who were in SD or PD at first restaging did not improve their status at the end of therapy. Globally, the median number of cycles administered was 6 (range 1-16).

**Figure 1:**
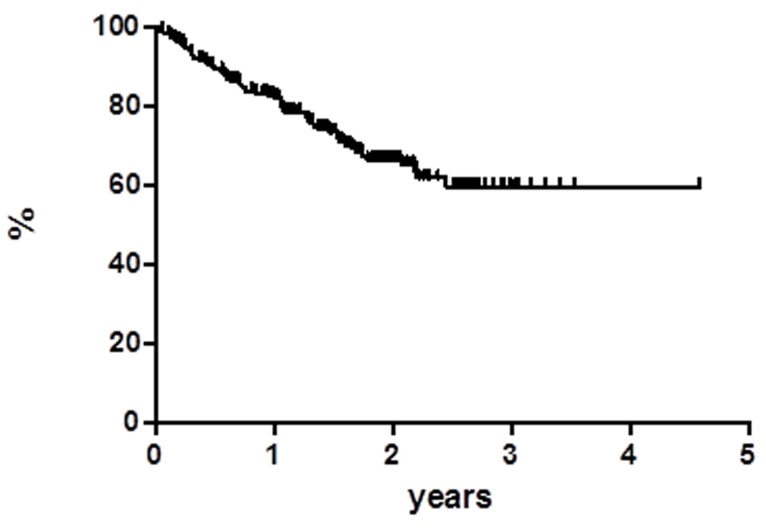
Overall survival

**Figure 2:**
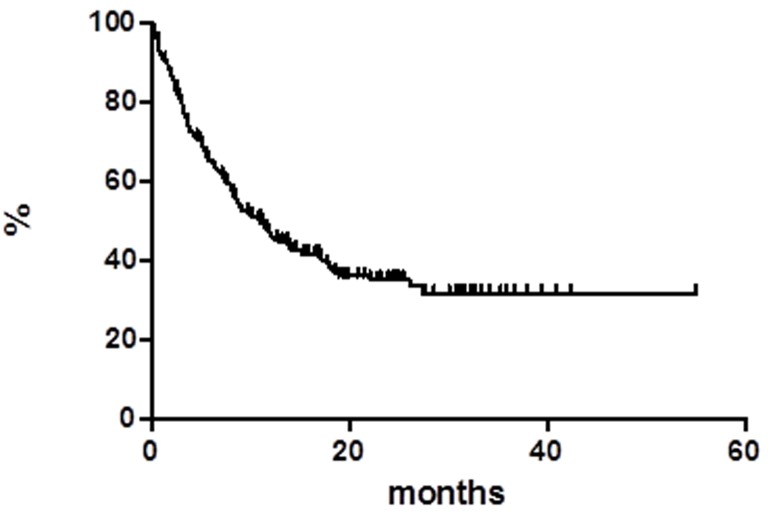
Progression free survival

**Figure 3:**
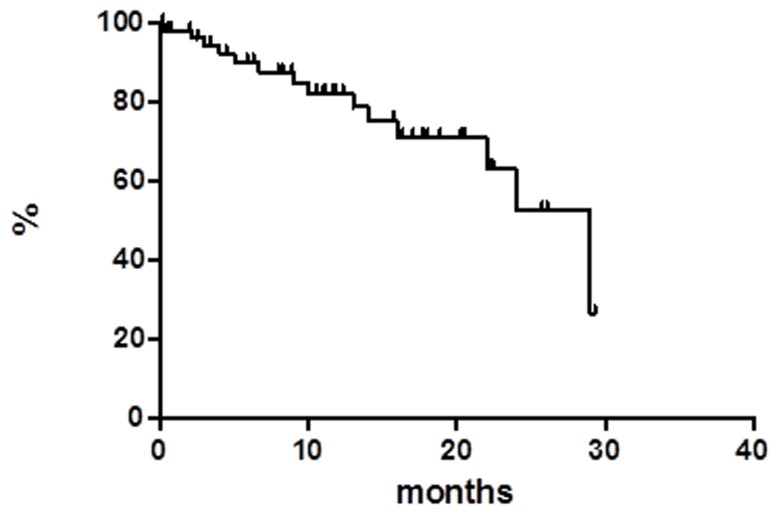
Disease free survival

At a median follow up of 18 months OS was 59.8% at 55 months (Figure 1), median has not reached yet. Global progression free survival (PFS) at 55 months was 31.9%, the median was achieved at 11 months (Figure 2). Global disease free survival (DFS) was 26.3% at 29 months (Figure 3); 14 out of 62 (22.6%) CR patients relapsed, whereas 48 patients are in continuous CR (CCR) with median duration of response (DoR) of 23.8 months.

**Figure 4:**
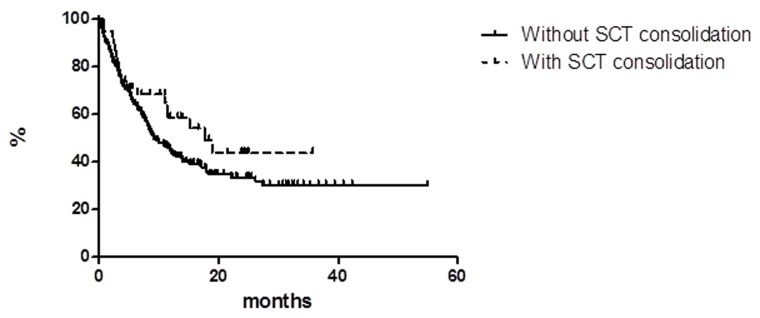
Progression free survival in patients with and without stem cell transplant (SCT) consolidation

Among the responder patients (CR + PR), 38 patients had a consolidation with transplant (16 ASCT and 22 alloSCT) next to BV treatment; the estimated PFS at 36 months as a function of transplant consolidation did not show statistically significant difference between patients with consolidation (43.7%, median reached at 17.6 months) vs. patients who did not undergo consolidation (30.1, median reached at 9.0 months) (Figure 4). Currently, there are 18/30 long term responder patients (LTR) still in CCR: 7 with consolidative transplant and 11 without any consolidative procedure. At the latest follow-up, 172 (73.5%) patients were alive and 62 deceased (47 due to lymphoma, 14 for other reasons, and 1 for acute myeloid leukemia [AML] at 2 months from the end of the treatment).

### Safety

All patients who received at least one BV infusion were included in the safety analysis. In general, the treatment was well tolerated and the toxicity profile was very similar to the previously published data. One-hundred-five (44.8%) patients had an early treatment discontinuation: 10 due to extrahematologic toxicity, 38 for bridge to transplant, and 57 due to PD, with 3 deaths. Globally only 15 (6.4%) patients performed 16 cycles.

Sixty-nine (29.5%) patients had at least one toxicity. Among hematologic side effects, grade 3/4 adverse events (AE) were reported in 9 patients: neutropenia (*n* = 7), thrombocytopenia (*n* = 1), and pancytopenia (*n* = 1) all related to BV. Grade 3/4 extrahematologic toxicity was observed in 22 patients: 1 pancreatitis (related), 2 respiratory failures (related), 1 AML, 1 acute respiratory distress syndrome and 17 peripheral sensory neuropathies (related). Resolution or improvement of peripheral neuropathy was observed in 90% of patients with a median time to resolution or improvement of 12 weeks.

## DISCUSSION

This retrospective multicenter Italian study on 234 patients with R/R HL represents one of the two larger reports ever published together with the French experience.[18] To note, in the French report were included also 37/240 (15.4%) patients who had allo-SCT prior to BV. Our results are in accordance to the pivotal phase II study and to the other experiences both experimental and observational with an ORR of 59.8% and a CR rate of 31.6% in terms of best response, i.e. achieved any time during treatment.[11, 12, 14, 16-18, 23, 24]

Previous published data refer to a NPP. There is a boundary zone in the passage from phase III to phase IV trials, i.e. from experimental to marketing and free use phases: in this zone patients can be treated with unapproved drugs by means of NPP, compassionate and off-label use and, in Italy as in the present report, under request according to Law 648/96. The main difference between a NPP and Law 648/96 lies in inclusion/exclusion criteria: under Law 648/96 physicians use the drug in a wider spectrum of patients with varied underlying diseases and a broad range of concomitant medications. Thus, we added additional useful information about the management of BV in the real life.

No selection bias at all, as BV was prescribed as per clinical indication and Centers and patients were consecutively enrolled.

Some interesting considerations about the role of BV in everyday clinical practice can be extrapolated. The best response rate was higher in the elderly setting (28 patients with an age >60 years) with an ORR of 67.8% and a CR rate of 50% (all in CCR at the latest follow-up) confirming the high activity of BV in this patients subset at the same number of cycles performed [24, 25]. The comparison with the report summing up BV clinical trials results between 2006 and 2012 shows that in our series the clinical response is superior in terms of CR, ORR, and CCR.[13, 24] The patients who had a prior ASCT (suggesting chemo-sensitivity) had a better ORR than those who had not (suggesting chemo-resistance and primary refractory disease), while CR rates are comparable. This observation provides additional data on the activity of BV in refractory patients.

To be in CR after 4 cycles is confirmed very important for classifying the patient as a real good responder; at the same time, the right number of cycles to be performed for evaluating the potential consolidation with transplant (in the major part allogeneic transplant) or the continuation with BV until the cycle 16 remains an open issue, mainly because in case of CR the choice between the two options is at the physician discretion. Recently, Chen et al., updating on the pivotal phase II study, reported that the 5-year PFS was 67% in CR patients submitted to allotransplant next to BV versus 48% in patients who continued treatment after achieving CR to BV at the first restaging without any statistically significant difference.[11, 22] Moreover, in this update the authors reported that 9% of the enrolled study population has achieved a long-term remission (exceeding 5 years) without any further anti-lymphoma therapy after treatment with single agent BV. In our study, the estimated PFS at 3 years did not show statistically significant difference between patients who underwent consolidation (43.7%) vs. patients who did not (30.1%). At the time of writing, 48 (20.5% of all study population) patients were in CCR with a median duration of response of 29 months. Thus, also in the real life experience these survival outcomes showed that a substantial subset of patients among R/R HL obtained CR to single agent BV with a long-term disease control. Also the length of the follow up denotes the potential cure in some patients despite their lymphoma history.

An important question remains unanswered: among the CR patients, which may benefit from the transplant consolidation? In our series there were 30 LTR patients and 18 are still in CCR, 7 with consolidative transplant next to BV and 11 without any consolidative procedure: among these two subsets it was impossible to extrapolate any significant clinical characteristic to identify which is the best profile to move to a transplant procedure. Our study indicated that for patients who obtained a SD or PD after 4 cycles the potential conversion rate to PR or CR with further BV administrations is close to zero: only one patients out of 84 (1.2%) achieved a CR after a SD. The final message is that when patients show SD or PD at first restaging, they have to be shifted rapidly to another treatment. On the other hand, for patients who achieved PR after first restaging it could be important to continue the treatment: in our series 15/66 (22.7%) patients showed a conversion from PR to CR status, suggesting they continued to benefit from therapy.

Another interesting issue is the use of BV as a bridge to ASCT for chemorefractory patients: literature data in this topic show great promise.[24, 26, 27] In our study 16 patients were treated with BV before ASCT due to chemorefractoriness and were intended to proceed to ASCT. Among them, 8 patients had a response and finally 8 patients underwent ASCT. The toxicity profile was closely similar to the previously published data; no death has been linked to BV-induced toxicity.

In conclusion, the results of this large retrospective study on 234 R/R HL in the real world support the effectiveness of BV with a manageable toxicity as previously reported also in clinical trials; in particular, our report confirms activity also in elderly patients, duration of the clinical response unrelated to the consolidation with transplant procedure, the relevance of the CR status at first restaging for the quality of the final response, and the role of BV as a bridge to ASCT for chemorefractory patients.

## MATERIALS AND METHODS

An observational retrospective study was conducted on patients with R/R HL treated with BV in 40 Italian centers outside clinical trials.[28] The study was approved by our institutional board (Azienda Ospedaliera di Bologna, Policlinico S.Orsola-Malpighi, coordinating center) and by all involved Ethical Committees and registered in the Italian Registry of Observational Studies. All participants gave written informed consent in accordance with the Declaration of Helsinki. A shared database was used after the approval of all the co-investigators and variables were strictly defined to avoid bias in reporting data. We obtained a special permission (for scientific purpose) from our Ethical Committee to collect even data of patients who were deceased or lost to follow up.

From November 2012 to July 2014, a total of 234 patients with R/R HL were treated with BV according to the Italian law 648/96. All patients had histologically confirmed CD30+ disease; subjects were relapsed after prior ASCT or relapsed after at least two lines of chemotherapy if not eligible for ASCT due to insufficient stem cell collection or chemorefractory disease.

Patients received a 30-minute infusion of BV at the dose of 1.8 mg/kg of body weight every 3 weeks for a maximum of 16 cycles. Dose reduction to 1.2 mg/kg was recommended in case of grade 3 toxicity. The treatment was interrupted in case of grade 4 toxicity.

The primary endpoint of the study was the best response achieved any time during BV therapy; secondary endpoints were ORR at the end of the treatment, DoR, OS, PFS, DFS, and the incidence and severity of any AE occurring during treatment. Effectiveness was also evaluated through the occurrence of LTR patients, defined as patients who have response (CR or PR) duration ≥12 months. Response was assessed by PET/CT scan after cycle 4, 8, 12 and at drug discontinuation using the International Working Group revised response criteria for malignant lymphoma.[29] Safety and tolerability were evaluated by recording incidence, severity, and type of any AE according to the National Cancer Institute Common Terminology Criteria for AEs v4.0.

ORR was defined as the sum of CR and PR rates at the end of BV treatment and before any type of consolidation. OS was defined as the time from initiation of therapy to death from any cause and was censored at the date of last available follow up. PFS was measured from initiation of therapy to progression, relapse, or death from any cause and was censored at the date of last available follow up. DFS was calculated for CR patients from the first documentation of response to the date of relapse or death due to lymphoma or acute toxicity of treatment. DoR was calculated from the first objective tumor response (CR or PR) to first documentation of progression or death.[29]

Demographics and patients’ characteristics as well AEs were summarized by descriptive statistics. Survival functions were estimated by using the Kaplan-Meier method and were compared using log-rank test. Statistical analyses were performed with Stata 11 (StataCorp LP, TX) and p values were set at 0.05.

## Abbreviations

AE: adverse event
alloSCT: allogeneic stem cell transplant
ASCT: autologous stem cell transplant
BV: brentuximab vedotin
CCR: continuous complete response
CR: complete response
DFS: disease free survival
DoR: duration of response
HL: Hodgkin’s lymphoma
LTR: long term responder
ORR: objective response rate
OS: overall survival
PD: progression of disease
PR: partial response
PFS: progression free survival
R/R: relapsed/refractory
SD: stable disease
